# A novel approach to attention mechanism using kernel functions: Kerformer

**DOI:** 10.3389/fnbot.2023.1214203

**Published:** 2023-08-24

**Authors:** Yao Gan, Yanyun Fu, Deyong Wang, Yongming Li

**Affiliations:** ^1^Information Science and Engineering Department, Xinjiang University, Ürümqi, China; ^2^Beijing Academy of Science and Technology, Beijing, China; ^3^Key Laboratory of Big Data of Xinjiang Social Security Risk Prevention and Control, Xinjiang Lianhai INA-INT Information Technology Ltd., Ürümqi, Xinjiang, China

**Keywords:** linear attention, kernel method, transformer, SE Block, self-attention

## Abstract

Artificial Intelligence (AI) is driving advancements across various fields by simulating and enhancing human intelligence. In Natural Language Processing (NLP), transformer models like the Kerformer, a linear transformer based on a kernel approach, have garnered success. However, traditional attention mechanisms in these models have quadratic calculation costs linked to input sequence lengths, hampering efficiency in tasks with extended orders. To tackle this, Kerformer introduces a nonlinear reweighting mechanism, transforming maximum attention into feature-based dot product attention. By exploiting the non-negativity and non-linear weighting traits of softmax computation, separate non-negativity operations for *Query*(*Q*) and *Key*(*K*) computations are performed. The inclusion of the SE Block further enhances model performance. Kerformer significantly reduces attention matrix time complexity from *O*(*N*^2^) to *O*(*N*), with *N* representing sequence length. This transformation results in remarkable efficiency and scalability gains, especially for prolonged tasks. Experimental results demonstrate Kerformer's superiority in terms of time and memory consumption, yielding higher average accuracy (83.39%) in NLP and vision tasks. In tasks with long sequences, Kerformer achieves an average accuracy of 58.94% and exhibits superior efficiency and convergence speed in visual tasks. This model thus offers a promising solution to the limitations posed by conventional attention mechanisms in handling lengthy tasks.

## 1. Introduction

The Transformer model and its variants have emerged as state-of-the-art approaches in various Artificial Intelligence (AI) tasks, including natural language processing (Devlin et al., 2018), computer vision (Carion et al., 2020; Dosovitskiy et al., 2020), and audio processing (Baevski et al., 2020), demonstrating impressive performance across a wide range of benchmarks. As evident from the Transformer model and its variants, researchers are continually exploring new methods and extensions to tackle challenges in different AI tasks, leading to remarkable achievements. For instance, in the field of speech emotion recognition, some works (Kakuba et al., 2022a,b) have made improvements to attention mechanisms, highlighting the widespread application and significance of Transformers and their extensions in diverse domains.

The core component of the Transformer is its attention mechanism, which efficiently encodes contextual information by modeling correlations between different positions in the input sequence. However, the original self-attention mechanism in the Transformer model, relying on dot product similarity, has limitations in modeling complex and non-linear relationships among tokens, and exhibits quadratic computational complexity concerning sequence length. Consequently, traditional Transformer models encounter challenges in handling long sequence data, particularly in terms of computational complexity and position information processing. Our approach aims to address this by reducing the time complexity of the attention matrix while maintaining accuracy in processing NLP tasks.

To overcome these challenges, researchers have proposed various extensions, including low-rank approximations, sparse patterns, and locality-sensitive hashing. Nevertheless, these methods still rely on dot product similarity and may not adequately capture diverse relationships among tokens. Recently, kernel methods have been introduced to enhance Transformer efficiency, allowing clever mathematical re-writing of the self-attention mechanism to avoid explicit computation of the N × N matrix.

In this paper, we propose a novel self-attention mechanism called Kerformer, which utilizes kernel functions to redefine the attention mechanism and extract richer positional information through reweighting. We conducted experiments on NLP and CV tasks, showing that Kerformer outperforms the original self-attention mechanism and other extensions in terms of accuracy and computational efficiency. Additionally, we performed an ablation study to analyze the impact of different kernel functions and reweighting positions on Kerformer's performance.

In comparison to state-of-the-art methods in self-attention and transformer architectures, our proposed Kerformer introduces a novel and efficient approach to self-attention computation. While previous works, such as Linformer (Wang et al., 2020), Reformer (Kitaev et al., 2020), DCT-Former (Scribano et al., 2023), LISA (Wu et al., 2021), and Bernoulli sampling attention mechanism (Zeng et al., 2021), have made significant strides in reducing computational costs and improving efficiency, they still rely on dot product similarity and may have limitations on sequence length and global dependencies.

In contrast, Kerformer leverages kernel methods to redefine the attention mechanism, enabling the capture of more complex and non-linear relationships among input tokens. By applying a kernel function and SE Block module to the concatenation of query and key vectors, Kerformer computes attention weights using the resulting kernel matrix, thereby modeling various types of relationships with enhanced expressiveness.

Moreover, our Kerformer introduces reweighting mechanisms that extract richer positional information, addressing challenges in long sequence processing and enhancing computational efficiency. This combination of kernel-based self-attention and reweighting sets Kerformer apart from existing approaches, making it a promising extension to the transformer architecture.

In the upcoming sections, we analyze existing self-attention methods and their limitations. We introduce the Kerformer model, discussing its novel kernel-based self-attention and reweighting mechanisms. We present experimental results and compare Kerformer with state-of-the-art methods on NLP and CV tasks. Finally, we discuss implications and conclusions in self-attention modeling.

In summary, our study introduces a novel self-attention mechanism, Kerformer, which utilizes compute kernels and reweighting techniques to capture intricate and diverse token interactions, while effectively addressing the computational complexity associated with long sequence tasks. By reducing the attention matrix complexity without compromising accuracy, Kerformer demonstrates its efficacy in various NLP and CV applications. Our research findings contribute to the advancement of more expressive and efficient self-attention mechanisms.

## 2. Related work

Self-attention has become a fundamental building block of modern neural architectures in natural language processing and computer vision. The original transformer architecture introduced by Vaswani et al. (2017) utilized self-attention as a key component to compute the representation of each input token. Since then, numerous variants of the transformer architecture have been proposed to overcome various limitations, such as the lack of position information and the quadratic complexity with respect to the sequence length.

Efforts have been made to improve the efficiency of self-attention, with several methods proposed to reduce computation costs. These include the Linformer (Wang et al., 2020), which approximates the self-attention matrix with a low-rank matrix, and the Reformer (Kitaev et al., 2020), which introduces locality-sensitive hashing to accelerate self-attention computation. DCT-Former (Scribano et al., 2023) achieves efficient self attention computation by introducing discrete cosine transform as a frequency domain based conversion method. By calculating attention weights in the frequency domain, DCT-Former can significantly reduce computational complexity while maintaining high performance, improving the efficiency and scalability of the model. LISA (Wu et al., 2021) utilizes a codeword histogram technique to achieve linear-time complexity for self-attention computation. By representing tokens as codewords and constructing histograms based on their frequencies, the model efficiently captures token interactions and calculates attention weights. This approach reduces the computational overhead associated with traditional self-attention mechanisms, making it suitable for large-scale recommendation tasks. A Bernoulli sampling attention mechanism (Zeng et al., 2021) based on locally sensitive hashing (LSH) approximates the calculation of self attention weights through random sampling, thereby reducing computational complexity to a linear level. The Bernoulli sampling method can significantly reduce the time and space overhead of self attention computation while maintaining good performance. However, the above methods often have limitations on the length of the sequence and limit the global dependencies of the sequence.

In addition, there are attempts to extend self-attention beyond its original formulation. For example, the Sparse Transformer (Child et al., 2019; Beltagy et al., 2020; Zaheer et al., 2020) introduces sparsity patterns to reduce computational costs. The Performer (Choromanski et al., 2020) uses an approximation of the softmax function to compute self-attention more efficiently. Moreover, Katharopoulos et al. (2020) reformulated the attention mechanism in the autoregressive Transformer model to use sequential computation, thereby reducing computation time and storage requirements. Nyströmformer (Xiong et al., 2021) proposed a method based on Nyström approximation, which approximates the calculation of self attention weight by decomposing the self attention Matrix decomposition into the product of low rank matrix. Nevertheless, these approaches may also exhibit certain limitations, including elevated memory usage, potential degradation of model accuracy, or approximation errors.

Recently, kernel-based methods have emerged as a promising extension of self-attention. Kernel methods replaces the dot-product similarity used in self-attention with a kernel function, allowing it to capture more complex interactions between input tokens and enabling the use of more powerful kernel functions to model various types of relationships. This method allows iterative implementation, which significantly accelerates Transformer and reveals their relationship with recurrent neural networks. The Kernel methods mechanism has been successfully applied to various tasks, such as text classification and image classification. Skyformer (Chen et al., 2021) proposes a novel approach that employs a Gaussian kernel and the Nyström method to approximate self-attention, thereby reducing computational complexity while maintaining accuracy. This work shows promising results on several natural language processing tasks, including text classification and machine translation. Kernel self-attention (Rymarczyk et al., 2021) proposes a novel approach for weakly-supervised image classification by combining kernel self-attention with deep multiple instance learning. The method uses a kernel function to capture complex interactions between image regions and enable more powerful modeling of relationships.

Several modifications to attention have been proposed by researchers, including the use of softmax to operate *Q* and *K* matrices separately (Bhandare et al., 2019), and the decomposition of attention into kernel functions, with *Q* and *K* matrices operated on using the *elu* and *relu* functions, respectively (Katharopoulos et al., 2020; Qin et al., 2022). These modifications reduce the complexity of attention from *O*(*N*^2^) to *O*(*N*), which is beneficial for large-scale models.

In comparison to the state-of-the-art methods in self-attention and transformer architectures, our proposed Kerformer introduces a novel and efficient approach to self-attention computation. While previous works, such as Linformer, Reformer, DCT-Former, LISA, and Bernoulli sampling attention mechanism, have made significant strides in reducing computational costs and improving efficiency, they still rely on dot product similarity and may have limitations on sequence length and global dependencies. In contrast, Kerformer leverages kernel methods to redefine the attention mechanism, enabling the capture of more complex and non-linear relationships among input tokens. By applying a kernel function and SE Block module to the concatenation of query and key vectors, Kerformer computes attention weights using the resulting kernel matrix, thereby modeling various types of relationships with enhanced expressiveness.

Moreover, our Kerformer introduces reweighting mechanisms that extract richer positional information, addressing challenges in long sequence processing and enhancing computational efficiency. This combination of kernel-based self-attention and reweighting sets Kerformer apart from existing approaches, making it a promising extension to the transformer architecture.

In conclusion, self-attention has undergone significant developments since its introduction in the original transformer architecture, with research focusing on improving its efficiency, scalability, and expressiveness. Kernel methods is a recent extension that shows promise in modeling complex relationships between input tokens, and several modifications have been proposed to enhance its performance. The Kerformer proposed in this study addresses the existing research gap by introducing kernel functions and reweighting mechanisms, effectively tackling challenges in long sequence processing and enhancing computational efficiency. The main idea of Kerformer is to change the order of operations of matrices according to the union law of matrices, so as to linearize the attention. When linearizing the attention, we first activate the Q and K matrices through the activation function to ensure the non-negativity of the attention matrix, and then reweight the K matrix through the SE-K module to achieve the redistribution of attention, so as to improve the performance of the model.

## 3. Methodology

In this section, we propose a novel linear Transformer model called **Kerformer**. We introduce a decomposable linear attention mechanism that replaces traditional softmax attention, resulting in improved time and memory complexity. Our method is also applicable to casual attention. The **Kerformer** model also employs different activation functions for *Q* and *K*, and combined with SE Block to reweight the activated *K*, which contributes to its faster computing speed and better performance.

### 3.1. Transformer

Given an input sequence *x* of length *N* and feature dimension *d*, we represent it as *x*∈ℝ^*N*×*d*^. The Transformer model can be formulated as Eq. 1.


(1) T(x)=F((A(x)+x)

In the Transformer model, the *F* implementation typically corresponds to a feedforward neural network that transforms the characteristics of each input. The attention function is denoted by *A*, and its time and memory complexity scales quadratically with respect to the input sequence length *N*.

The core idea of the attention mechanism is that the network should give different importance to different parts of the input data. When processing the input data, the network needs to assign different weights to different parts of the input in order to better capture the important information in the input data. This process of weight assignment is the attention mechanism.

In implementing the attention mechanism, two key components are usually used: *query*(*Q*), *key*(*K*), and *value*(*V*). A query is a vector in the network that represents the network's attention to the input data. Keys and values are vectors in the input data used to represent different parts of the input data. The attention mechanism achieves attention to the input data by computing the similarity between the query and the key and assigning weights to the values based on the similarity.

Regarding the attention function A, it consists of three essential components, including *query*(*Q*), *key*(*K*), and *value*(*V*). These components are computed from the input sequence *x* and three learnable matrices *W*_*Q*_, *W*_*K*_, and *W*_*V*_, respectively, as follows: *Q* = *xW*_*Q*_, *K* = *xW*_*K*_, *V* = *xW*_*V*_.

The final output *A* = *V*′ is obtained through a softmax function applied to *QK*^*T*^ line by line, which can be expressed as follows in Eq. 2.


(2) $$\begin{array}{l} A\left(x\right)=V^{\prime }=softmax\left(\frac{QK^{T}}{\sqrt{D}}\right)V \end{array}$$

We can interpret Eq. 2 as a specific instance of the attention mechanism, where the softmax function is applied to calculate *QK*^*T*^. In order to introduce a more generalized expression of attention, we can use *V*_*i*_ to represent the i-th row of a matrix *V*(*V*∈ℝ^*N*×*d*^). The equation of the generalized attention mechanism is shown below as Eq. 3. Similar derivations have been done in these works (Qin et al., 2022).


(3) $$V_{i}^{'}=\sum_{j=1}^{N}\frac{sim(Q_{i},K_{j})}{\sum_{j=1}^{N}sim(Q_{i},K_{j})}V_{j}$$

It should be noted that the function sim in Eq. 3 can be any correlation function that satisfies certain requirements, which will be explained later. If we choose $sim\left(Q,K\right)=e^{\frac{QK^{T}}{\sqrt{d}}}$, then Eq. 3 is equivalent to Eq. 2.

### 3.2. Linear attention

To maintain the linear computation budget, one feasible solution is to expand the *sim* function in the form of a kernel function, as shown in Eq. 4.


(4) $$\begin{array}{l} sim\left(q_{i},k_{j}\right)=\phi {\left(q_{i}\right)}^{T}\varphi \left(k_{j}\right) \end{array}$$

In Eq. 3, ϕ and φ are kernel functions used for the nonlinear mapping of queries and keys. We can rewrite Eq. 3 as a kernel function, as shown in Eq. 5.


(5) $$\begin{array}{l} V_{i}=\frac{\sum_{j=1}^{N}(\phi (Q_{i})\varphi {\left(K_{j}\right)}^{T})V_{j}}{\sum_{j=1}^{N}\left(\phi (Q_{i})\varphi {\left(K_{j}\right)}^{T}\right)} \end{array}$$

Then, the attention operation under linear complexity can be realized through the multiplication combination law of matrix, as shown in Eq. 6.


(6) $$\begin{array}{l} V_{i}=\frac{\phi (Q_{i})\sum_{j=1}^{N}\varphi {\left(K_{j}\right)}^{T}V_{j}}{\phi (Q_{i})\sum_{j=1}^{N}\varphi {\left(K_{j}\right)}^{T}} \end{array}$$

**Figure 1:**
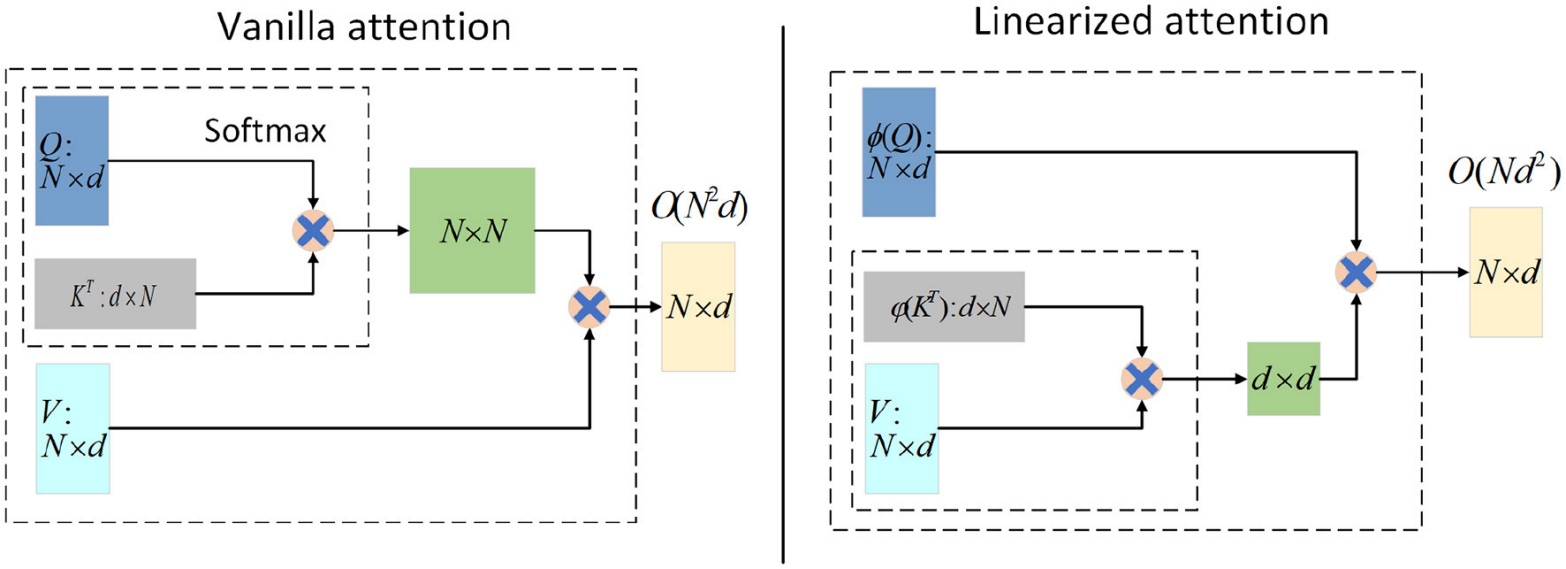
Illustration of the computations for Vanilla attention **(left)** and Linearized attention **(right)**. For input, the input length is *N* and the feature dimension is *d*. ϕ and φ represent the kernel function form for processing *Q* and *K*. Generally speaking, *d*≪*N*, Linearized attention can be approximately regarded as the time and memory complexity of *O*(*N*).

Note that in Eq. 4, the functions ϕ and φ are applied row by row to the matrices *Q* and *K*. By using the associative law of multiplication, *QK*^*T*^∈ℝ^*N*×*N*^ is calculated as φ(*K*)^*T*^*V*∈ℝ^*d*×*d*^. The result is then left multiplied by ϕ(*Q*)∈ℝ^*N*×*d*^, which represents the attention weights. This computation mode achieves a complexity of *O*(*Nd*^2^) for the attention mechanism. However, for long sequences where *d*≪*N*, the complexity can be considered as *O*(*N*), greatly reducing the overhead. This is illustrated in Figure 1.

### 3.3. Kerformer

The softmax operation applied in the attention mechanism is used to normalize the query and key matrices. However, there is no clear explanation for why the softmax operation is effective, and it is more of an empirical observation that leads to good model performance. Our aim is to enhance the attention mechanism by using the kernel form. Specifically, we want to generalize the attention mechanism using the kernel function and provide a theoretical foundation for the application of different operations in the attention mechanism. This will help us better understand the working principles of the attention mechanism and improve its performance.

Cosformer (Qin et al., 2022) discussed that the choice of ϕ and φ functions is crucial for the performance of attention mechanisms in kernel form. They proposed two empirical constraints that may play a significant role in achieving better performance:

(i) Non-negative constraint on the attention matrix to ensure that the attention weights are always positive and the attention is focused only on relevant features.

(ii) A nonlinear weighted scheme to focus attention on specific regions of the matrix distribution, which can capture more complex and subtle patterns.

It is worth noting that similar kernel function methods have been used to modify the attention mechanism in the works of Angelos and Qin et al. These works always choose the same activation function for both the ϕ and φ functions. We decided to choose different ϕ and φ functions to enhance the model's global learning ability and generalization ability.

To ensure the two constraints mentioned above, we use sigmoid activation function for ϕ(*Q*) and softmax activation function for φ(*K*) instead of the original *softmax*(*QK*^*T*^) in our work. Thus, we define our functions as shown in Eq. 7 and Eq. 8.


(7) $$\begin{array}{l} \phi \left(x\right)=sigmoid\left(x\right) \end{array}$$

(8) $$\begin{array}{l} \varphi \left(x\right)=softmax\left(x\right) \end{array}$$

We substitute Eqs 7 and 8 into Eq. 6 to obtain Eq. 9, as follows:


(9) $$\begin{array}{l} V_{i}=\frac{sigmoid(Q_{i})\sum_{j=1}^{N}softmax{\left(K_{j}\right)}^{T}V_{j}}{sigmoid(Q_{i})\sum_{j=1}^{N}softmax{\left(K_{j}\right)}^{T}} \end{array}$$

**Figure 2:**
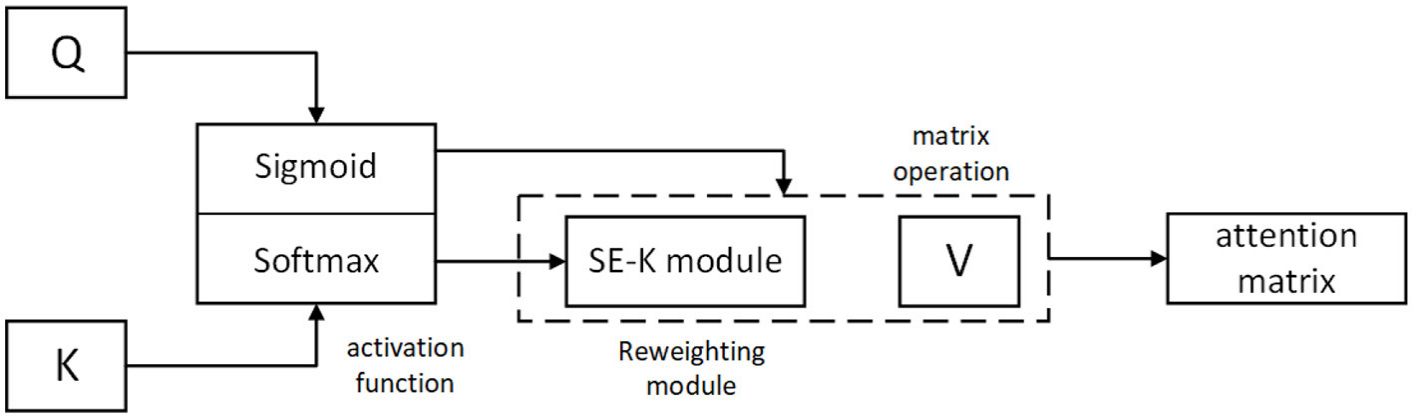
System block diagram of our approach Kerformer and workflow representation.

The system block diagram of Kerformer is shown in Figure 2.

### 3.4. Interpretation of Kerformer

Previous works, such as Katharopoulos et al. (2020) and Qin et al. (2022), have also rewritten self-attention in kernel form, but they have used the same function to transform both the *Q* and *K* matrices. The possible reason for this is that if different transformations are applied to the *Q* and *K* matrices, the relative positional relationship between them may be disrupted. This could lead to inaccurate score calculations and negatively affect the performance of the model.

However, Efficient attention (Shen et al., 2021) provided a new explanation for their proposed linear attention, which is different from self attention. They explained that linear attention does not generate attention maps for each position, and each ${\left(K_{j}\right)}^{T}$ is a global attention map that does not correspond to any position. Based on this explanation, we aim to introduce different functions for *Q* and *K* without disturbing the attention mechanism as much as possible, which may bring improvements to the model.

The explanation provided by Efficient attention (Shen et al., 2021) regarding linear attention inspired our work to introduce different functions for *Q* and *K* matrices. This would allow us to explore new explanations and extensions to the attention mechanism.

Our approach includes introducing different nonlinear mappings for *Q* and *K* matrices. We use the sigmoid operation on *Q* to limit its range between 0 and 1, mapping each element to a probability distribution. Similarly, we apply the softmax operation on *K* to also map each element to a probability distribution. This introduces more nonlinearity to the model, making it better suited to fit the data.

Furthermore, the model is forced to learn different information due to the effects of these operations. The sigmoid operation allows the model to focus more on keys that are similar to the query, while the softmax operation enables the model to focus more on elements with higher probabilities in the values. This combination allows the model to learn better in different directions.

Lastly, the use of the smooth sigmoid and softmax operations makes the model more robust to data disturbance or noise, reducing the risk of overfitting. Overall, our approach introduces new insights into the attention mechanism and improves the model's performance.

### 3.5. Reweighting of attention

The above explanation highlights the difference between linear attention and self-attention, with linear attention not generating attention maps for each position. Given this difference, we aim to introduce the SE module to perform re-weighting of the *K* matrix along the N dimension. The goal is to extract different features by using different functions for *Q* and *K* without disturbing the attention mechanism as much as possible, which could lead to improvements in the performance of the model. By using the SE module, we can dynamically recalibrate the feature maps of *K* based on their importance, thus improving the model's ability to extract meaningful information from the input data.

In order to adapt to the reweighting of the *K* matrix, we slightly modified the SE module and referred to it as the SE-K module. As mentioned earlier, the *K* matrix itself already possesses non-negative values, we remove the ReLU activation function from the SE module. The SE-K module is a modified version of the SE module that takes into account the non-negativity of the *K* matrix.

In this section, we will describe how we incorporate the SE-K module into the *K* matrix of the attention mechanism. Specifically, we apply the SE-K module to the N dimension of the *K* matrix, where K has a dimension of N x d.

The SE module is a simple yet effective mechanism that is widely used to enhance the representational power of neural networks. It selectively recalibrates the feature map by using the global information of the feature map. In our method, we use the SE-K module to recalibrate the K matrix, thereby improving its feature extraction ability.

**Figure 3:**
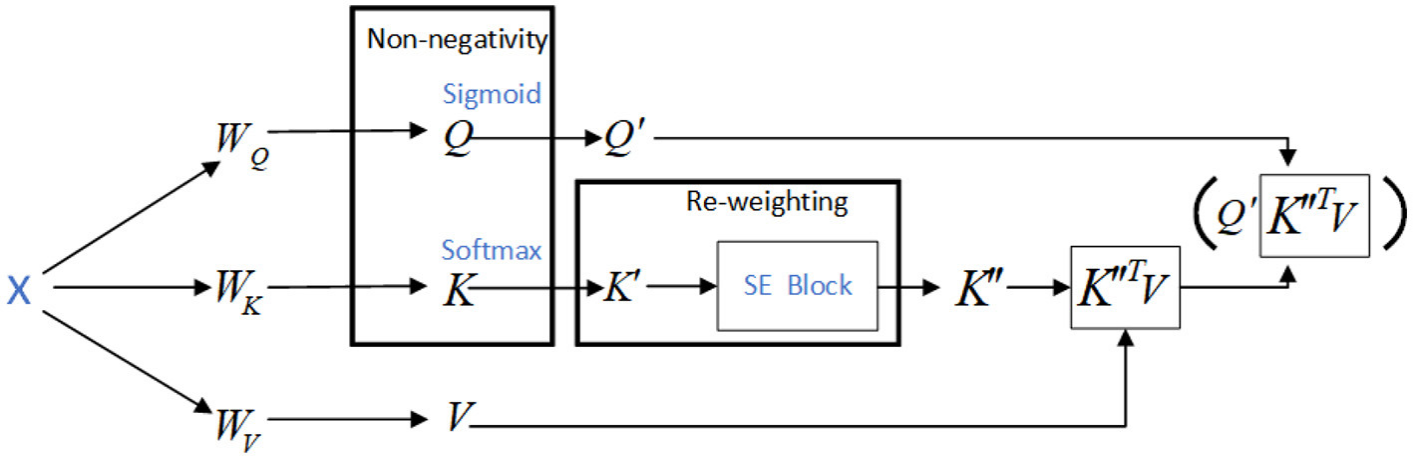
Use the activation functions *Sigmoid* and *Softmax* to activate the *Q* and *K* matrices respectively.

**Figure 4:**
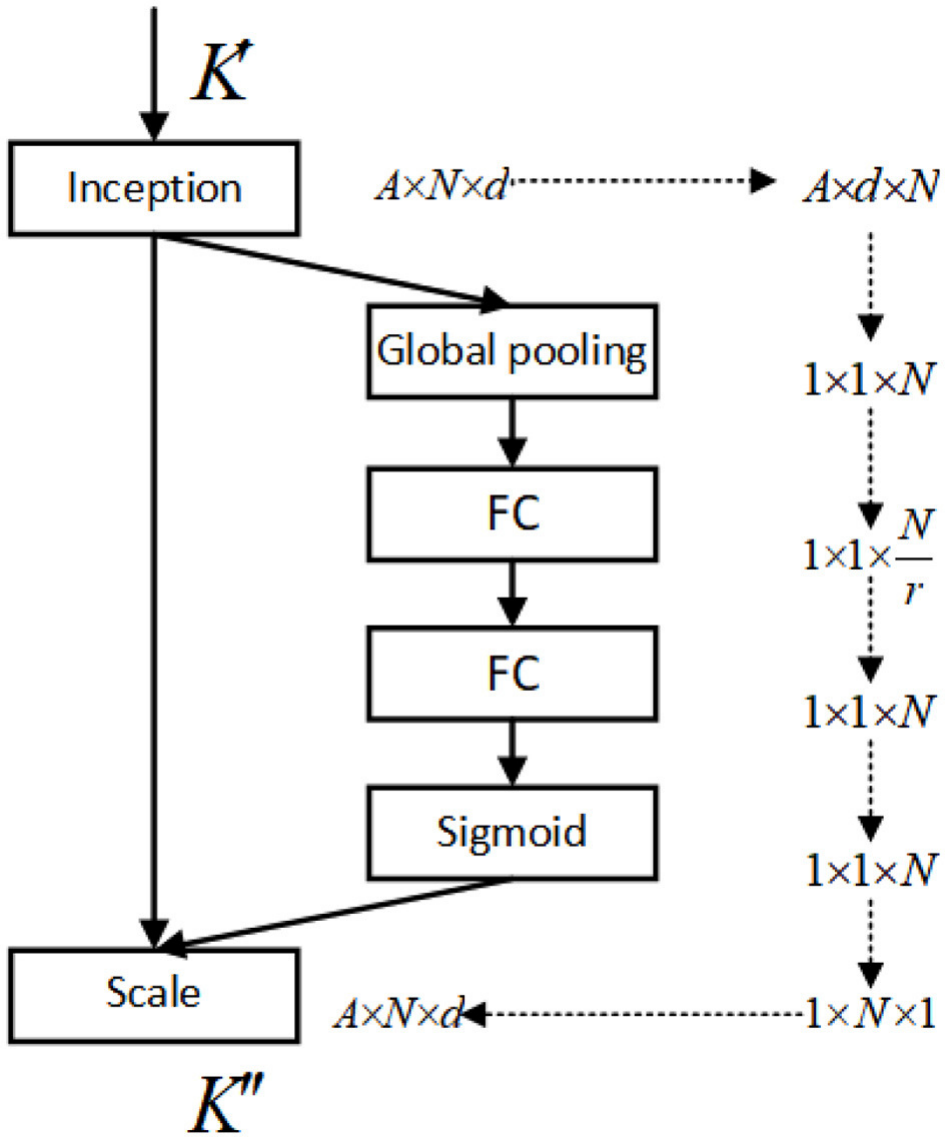
The structure of the SE-K module is shown, and the *K*′ matrix after activation by *Softmax* is reweighted.

To apply the SE-K module to the *K* matrix, we first perform a global pooling operation on the *K* matrix along the N dimension, resulting in a feature vector. This feature vector is then passed through two fully connected layers, which are followed by a sigmoid activation function. The output of the sigmoid function is a set of N-dimensional attention weights, which are used to weight the *K* matrix along the N dimension. Finally, the weighted *K* matrix is fed into the attention mechanism. The operation to activate the *Q* and *K* matrices is shown in Figure 3, and the network structure of the SE-K module involved is shown in Figure 4.

**Figure 5:**
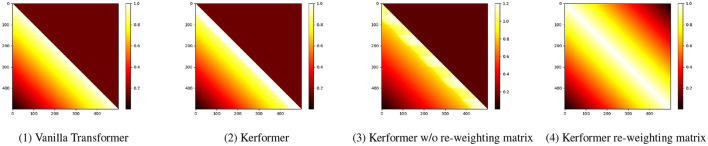
(1): Attention matrix of vanilla transformer.(2): Attention matrix of Kerformer.(3): Attention matrix of Kerformer without re-weighting. (4): Visualization of the re-weighting matrix.

For NLP tasks, Kerformer places more weight on neighboring tokens, thus enhancing locality. The weight distribution is shown in the Figure 5. By using the SE-K module, we can effectively learn the importance of different features in the *K* matrix, which can significantly improve the performance of the attention mechanism. Additionally, the SE-K module has a relatively small computational cost, which makes it easy to incorporate into existing neural network architectures.

Overall, our method of applying the SE-K module to the *K* matrix has shown promising results in various tasks, demonstrating its effectiveness in improving the feature extraction ability of the attention mechanism.

Our research method is based on the activation function and the reweighting mechanism. The activation function is to perform a non-negativity operation on the matrix to satisfy the requirement of non-negativity of the attention matrix, while the reweighting operation is to redistribute the attention weights to achieve the effect that the local influence on the nearby attention is greater. These two operations can better satisfy the attention relationship between different parts to obtain the final attention matrix. For data collection we use all the data sets that are now publicly available and conduct our experiments on these publicly available datasets.

## 4. Simulation experiments

In this section, we present an evaluation of our proposed method, Kerformer, through simulation experiments. The simulation experiment focuses on a mathematical evaluation of Kerformer. We compare our model with four baselines, Vanilla attention (Vaswani et al., 2017), Efficient attention (Shen et al., 2021), Linear-Elu (Katharopoulos et al., 2020), and Performer (Choromanski et al., 2020), to demonstrate the superiority of our approach in terms of model running memory, running time. All experiments were conducted using Matlab R2020a.

### 4.1. Comparison of time costs in simulation experiments

This experiment fixes the number of input matrices as 1 and the attention head dimension as 64, and compares the running time of each method by changing the sequence length size *N* of input *x*. The specific results can be seen in Table 1, with time units in seconds.

**Table 1:** Comparison of the time required to run the five methods for different methods in different dimensions of the input *x*, *Q*, *K*, and *V* in the case of taking values in the range [−2,2].

**Dimensional changes**	**Vanilla attention**	**Efficient attention**	**Linear-Elu**	**Performer**	**Kerformer(ours)**
1*1,000*64	4.001s	1.000s	1.000s	0.882s	0.200s
1*10,000*64	302.072s	31.015s	6.0121s	6.112s	5.852s
1*100,000*64	OOM	87.024s	51.014s	55.514s	44.011s
1*1,000,000*64	OOM	967.22s	506.134s	505.514s	521.144s

From the experimental results in Table 1, we can see that four other methods have a greater advantage over the Vanilla attention method in terms of the time cost of attention matrix computation, especially Vanilla attention has experienced memory overflow when the input sequence length *N* is large. In addition, our proposed method usually outperforms other methods with shorter computation time when the length of the input sequence *N* is below the million level. In practice, the model input length *N* is always below the million level. That is, our proposed method outperforms other methods in use.

From the experimental results in Table 2, it can be seen that four other methods have time cost advantages over Vanilla attention to different ranges of *Q*, *K*, and *V* values. Cosformer has more time cost advantage in computing Attention when the value range is [−10,10], while our method has a shorter running time compared to the other three methods for the range of values of *Q*, *K*, and *V* below [−10,10], which fully illustrates the advantage of our method in terms of time cost.

**Table 2:** Comparison of the time required to run the five methods with different ranges of values for *Q*, *K*, and *V* for different methods with the dimension size of the input *x* of 1*10,000*64.

**Range of values**	**Vanilla attention**	**Efficient attention**	**Linear-Elu**	**Performer**	**Kerformer(ours)**
[−1,1]	335.075s	34.007s	7.001s	6.854s	6.001s
[−2,2]	302.072s	31.015s	6.012s	6.112s	5.852s
[−4,4]	1,003.233s	35.008s	5.025s	6.012s	5.006s
[−6,6]	1,062.249s	34.008s	5.145s	5.541s	5.022s
[−8,8]	1,032.248s	35.993s	6.004s	6.125s	5.952s
[−10,10]	1,103.246s	55.013s	8.001s	7.854s	8.004s

### 4.2. Comparison of memory costs in simulation experiments

The experimental results in Table 3 show that the other four methods have a smaller memory consumption compared to the Vanilla attention method in the computation of the attention matrix. According to our empirical observation, the value range of *Q*, *K*, and *V* matrices input into the attention mechanism is mostly between [−4,4]. Our method has a memory cost advantage in the range of [−2,2] and [−4,4], which indicates that our method can achieve a low memory cost in the normal range of values, which can be attributed to the fact that our method uses different activation functions for *Q* and *K*, which can improve the computational speed and generalization ability of the model.

**Table 3:** Comparison of the memory requirements of the five methods running with different ranges of values for *Q*, *K*, and *V* for the input *x* with dimension size of 1*10,000*64.

**Range of values**	**Vanilla attention**	**Efficient attention**	**Linear-Elu**	**Performer**	**Kerformer(ours)**
[−1,1]	8,521M	521M	623M	689M	534M
[−2,2]	11,001M	585M	678M	702M	578M
[−4,4]	12,454M	623M	725M	754M	602M
[−6,6]	14,845M	685M	775M	801M	692M
[−8,8]	15,624M	725M	835M	833M	754M
[−10,10]	16,104M	785M	877M	892M	802M

## 5. NLP task

We empirically validate the effectiveness of our proposed Kerformer method in multiple aspects. Firstly, we examine its generalization capability on downstream tasks by comparing it with other existing transformer variants. Then, we conduct a comparison with other Long-range arena benchmark transformer variants to assess its ability to model long-range dependencies and to perform a thorough analysis of model efficiency.

### 5.1. Downstream fine-tuning tasks

First, we performed the Kerformer model and the remaining five models [Performer (Choromanski et al., 2020), Reformer (Kitaev et al., 2020), and Liner Trans (Katharopoulos et al., 2020), Longformer (Beltagy et al., 2020), RFA (Peng et al., 2021), and Dct-former (Scribano et al., 2023)] were compared in terms of accuracy. This was achieved by conducting comparative fine-tuning experiments on five datasets, including GLUE (QQP, SST-2, MNLI) (Wang et al., 2018), IMDB (Maas et al., 2011), and Amazon (Ni et al., 2019). In the experiments, pre-trained models are used and fine-tuned in the downstream text classification task, and the results are shown in Table 4. From Table 4, we can see that Kerformer fetches the best accuracy in addition to the baseline (Liu et al., 2019) on the QQP, SST-2 and IMDB downstream text classification tasks. Although Dct-former and Longformer achieved better classification accuracy than Kerformer on MNLI and AMAZON tasks, respectively. It has higher computational complexity compared to our method. This is related to Kerformer's activation of *Q* and *K* matrices with activation functions and reweighting of K matrices respectively, where the activation functions can extract features in the matrices and reweighting can effectively reallocate attention to achieve the effect of expanding local attention. The experimental result fully demonstrates the effectiveness of our proposed Kerformer model.

**Table 4:** Results of fine-tuning downstream tasks based on pretrained bidirectional models.

	**QQP ↑**	**SST-2 ↑**	**MNLI ↑**	**IMDB ↑**	**AMAZON ↑**	**Avg ↑**
Vanilla transformer	88.52	92.25	80.02	92.55	75.65	85.80
Performer	69.95	50.82	35.28	60.41	64.25	56.14
Reformer	63.12	50.66	35.35	49.88	64.32	52.67
Liner Trans	74.75	84.72	66.35	91.21	72.62	78.07
Longformer	85.55	88.56	77.27	91.07	**73.52**	83.13
RFA	75.32	76.44	57.71	78.86	68.08	71.28
Dct-former	85.56	86.89	**77.48**	89.68	72.12	80.19
**Kerformer**	**85.68**	**90.21**	76.32	**91.50**	73.24	**83.39**

Best results are shown in bold. Our proposed Kerformer shows superior performance compared to competing efficient transformers and is approaching vanilla transformers.

### 5.2. Long sequence experiment results

To assess the generalization performance of our proposed method Kerformer, we conducted training from scratch on the Long-range Arena benchmark 2020b. This benchmark is tailored for evaluating the performance of efficient transformers on long input sequences, making it an appropriate test platform for comparative analysis of different efficient transformer variants. We evaluated our approach on various tasks, including long sequence ListOps (Nangia and Bowman, 2018), byte-level text classification (Maas et al., 2011), document retrieval using ACL selection networks (Radev et al., 2013), and Pathfinder (Linsley et al., 2018). While comparing with our Kerformer model with Local Attention (Tay et al., 2020), Reformer (Kitaev et al., 2020), Performer (Choromanski et al., 2020), Longformer (Choromanski et al., 2020), Transformer (Vaswani et al., 2017), BigBird (Zaheer et al., 2020), and Dct-former (Scribano et al., 2023) models, the comparison results of the seven different models are shown in Table 5. As shown in Table 5, Kerformer obtained the best performance in ListOps, Document Retrieval, while Kerformer also achieved competitive results in the other two tasks, and finally Kerformer achieved the next best score in overall task average accuracy. This is a good indication of Kerformer's strength in the long-range arena.

**Table 5:** Long-range arena benchmark test results.

**Model**	**ListOps ↑**	**Text ↑**	**Retrieval ↑**	**Pathfinder ↑**	**Avg ↑**
Local attention	15.67	52.87	53.40	66.59	47.13
Reformer	37.32	56.12	53.42	68.47	53.83
Performer	17.96	65.45	53.79	**77.08**	53.57
Longformer	35.65	62.79	56.83	69.69	56.24
Transformer	36.42	64.37	57.52	71.42	57.43
BigBird	36.11	64.08	59.31	74.79	58.57
Dct-former	36.55	**65.15**	59.55	75.56	**59.20**
**Kerformer**	**36.95**	64.32	**59.98**	74.52	58.94

The best results are shown in bold and the second best results are underlined. Kerformer obtained the best average score in four different tasks.

### 5.3. Ablation experiments

To verify the effectiveness of our chosen activation function in combination with the SE-K module, we conducted ablation experiments on GLUE (QQP, SST-2) (Wang et al., 2018) and IMDB (Maas et al., 2011) in downstream fine-tuning tasks, ListOps (Nangia and Bowman, 2018) in Long sequence tasks, byte-level text classification (Maas et al., 2011) and document retrieval using ACL selection networks (Radev et al., 2013) were conducted for the ablation experiments, and the results of the experiments are shown in the following Table 6.

**Table 6:** Ablation experiments are performed for the SE Block in the downstream fine-tuning task and the long sequence task of the reweighting module.

**Model structure**	**QQP**	**SST-2**	**IMDB**	**ListOps**	**Text**	**Retrieval**
Q + Softmax(K) + SE-K	81.25	85.63	85.24	33.25	58.53	55.89
Sigmoid(Q) + K + SE-K	82.36	87.25	88.25	35.21	60.25	57.26
Sigmoid(Q) + Softmax(K)	81.26	85.09	85.18	32.23	57.87	56.31
Kerformer	85.68	90.21	91.50	36.95	63.32	59.98

As shown in Table 6, Q + Softmax(K)+SE-K indicates that no activation operation is performed on the *Q* matrix, Sigmoid(Q) + K + SE-K indicates that no activation operation is performed on the *K* matrix, and Sigmoid(Q) + Softmax(K) indicates that no reweighting operation is performed. Based on the results of the ablation experiments, it can be seen that the activation of the *Q* and *K* matrices and the reweighting operation on the *K* matrix can effectively improve the performance of the model in the downstream fine-tuning task and the long-sequence task relative to other methods, and the effectiveness of our method is also demonstrated.

### 5.4. Efficiency comparison

In addition to comparing model performance, we also compared the computational speed of the different models. We compared the computational speed of Kerformer with other models [standard Transformer (Vaswani et al., 2017), Local Attention (Tay et al., 2020), Reformer (Kitaev et al., 2020), BigBird (Zaheer et al., 2020), Linear Trans (Katharopoulos et al., 2020), Performer (Choromanski et al., 2020), Longformer (Beltagy et al., 2020), and Dct-former (Scribano et al., 2023)], and the variable for comparison was the length of the input sequence, and the results of the experiments are shown in Table 7. We used byte-level text classification benchmarks to measure the computational speed of different models during training and inference for different sequence lengths (1k–4k).

**Table 7:** Speed comparison in training and inference for long-range arena benchmarks with different sequence lengths (1–4k).

	**Inferrence speed (steps per second)**↑				**Train speed (steps per second)**↑			
**Model**	**1K**	**2K**	**3K**	**4K**	**1K**	**2K**	**3K**	**4K**
Transformer	25.42	7.85	\	\	6.91	2.19	\	\
Local attention	57.69	33.21	23.32	17.80	13.42	6.61	4.35	3.10
Reformer	44.23	21.60	12.75	8.35	11.60	5.01	2.96	1.97
BigBird	20.92	11.53	8.14	6.12	6.50	3.21	2.09	1.55
Linear Trans	67.81	38.22	26.30	19.92	11.88	5.56	3.54	2.49
Performer	74.20	42.35	29.53	22.43	14.23	6.50	4.13	2.93
Longformer	23.02	6.33	\	\	4.42	1.31	\	\
Dct-former	56.21	34.21	22.85	20.51	11.58	5.95	3.92	2.32
Kerformer	57.42	33.15	21.45	17.13	11.34	5.58	3.57	2.55

If a method runs out of memory, we mark it with a backslash. The higher it is, the better it is.

Our method Kerformer achieves good training and inference speeds on sequence lengths 2K, 3K, and 4K, which illustrates the advantage of our method for speed computation on long sequence let tasks. This is because first the *Q* and *K* matrices are activated, then the *K* matrices are reweighted separately, and finally the order of computation of the self-attentive matrices can be exchanged using the union law of matrices so that the goal of linear complexity can be achieved. In conclusion, our model Kerformer achieves better overall efficiency compared to other linear variables, while maintaining excellent modeling and generalization capabilities.

## 6. Visual classification task

By incorporating distinct functions into the *Q* and *K* matrices, Kerformer is specifically designed to facilitate feature extraction at different levels, which is highly advantageous for visual classification tasks. The primary objective of our study is to showcase the superior performance of Kerformer in such tasks. To achieve this, we conducted comprehensive image classification experiments to rigorously evaluate the effectiveness and efficiency of Kerformer.

In order to assess the performance of Kerformer in image classification tasks, we applied it to the widely-used ViT-B/16 (Dosovitskiy et al., 2020) model and compared its accuracy with that of several baseline models, including Vanilla attention (Vaswani et al., 2017), Efficient attention (Shen et al., 2021), Linear-Elu (Katharopoulos et al., 2020), and Cosformer (Qin et al., 2022). To this end, we evaluated the models on four datasets: MNIST, CIFAR-10, CIFAR-100, and the flower dataset provided by TensorFlow.

The MNIST dataset consists of handwritten digital images, consisting of 60,000 training images and 10,000 test images, each representing a gray number from 0 to 9. Cifar-10 is a widely-used computer vision dataset for object recognition, comprising 60,000 RGB color images with dimensions of 32 × 32 pixels, distributed across 10 different classes. CIFAR-100 dataset contains 100 classes, grouped into 20 superclasses. Each image in CIFAR-100 is labeled with a “fine” class (specific class) and a “coarse” class (superclass). The flower dataset includes images of daisies and encompasses five flower types: “daisy,” “dandelion,” “rose,” “sunflower,” and “tulip."

Overall, our results suggest that Kerformer has strong feature extraction ability and outperforms the baseline models in terms of accuracy.

### 6.1. Test accuracy

In this section, we performed accuracy tests on the image classification tasks using the aforementioned four datasets. For all datasets except the flower dataset, the experiments were conducted with the following settings: the images were resized to 224 × 224 pixels, Adam optimizer was employed, the learning rate was set to 0.0001, the loss function used was Cross Entropy, the batch size was set to 32, and the training was carried out for 180 epochs. The final test accuracy was computed by averaging the results of 10 test runs. Due to the limited size of the flower dataset, the experimental configuration differed in terms of a smaller batch size of 4, a reduced training epoch of 80, and the final test accuracy was determined by averaging the results of 10 test runs.

**Figure 6:**
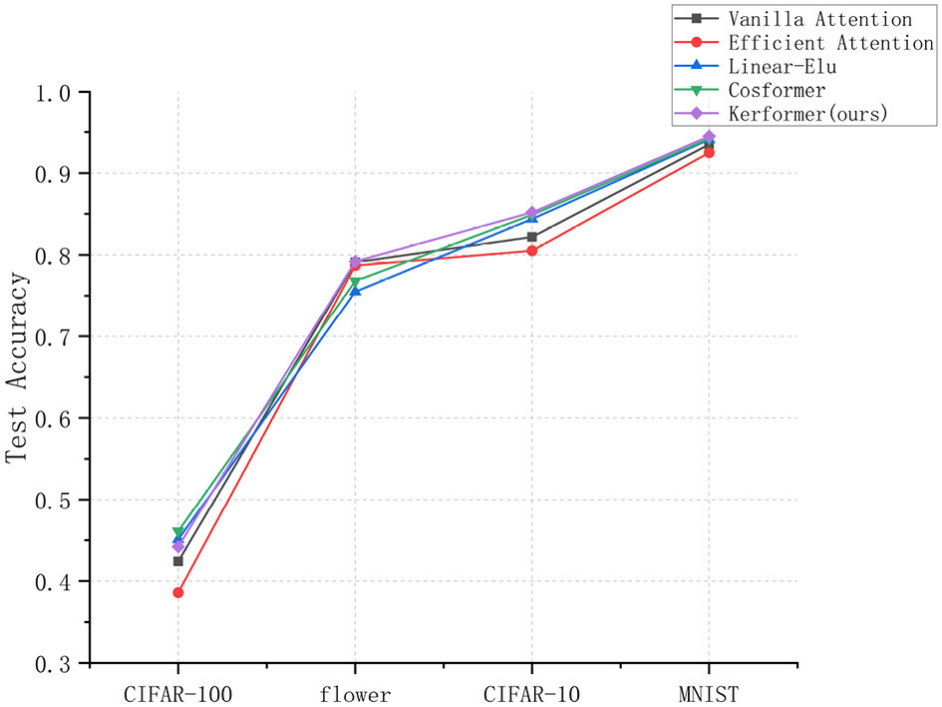
Experimental results of image classification accuracy measured by models using five methods (Vanilla attention, Efficient attention, Linear-Elu, Cosformer, and Kerformer) on different datasets.

Based on the experimental results shown in Figure 6, it is evident that the Cosformer method can achieve the highest model accuracy for image classification on the CIFAR-100 dataset, whereas our proposed method can achieve the highest test accuracy for image classification on the MNIST, CIFAR-10, and flower datasets. In particular, our method can improve 3% points compared to Vanilla attention method on CIFAR-10 dataset, which is a better test for the model performance improvement of the original model. Our results suggest that our proposed improvement can significantly enhance the performance of the model. In particular, this enhancement enables the model to more effectively utilize feature information from various locations, thereby improving its ability to extract essential features and ultimately increasing the classification accuracy of the model. This is due to the use of operations such as pooling in the SE-K module, which can perform better in image tasks because it is not limited by the global nature.

### 6.2. Convergence speed

**Figure 7:**
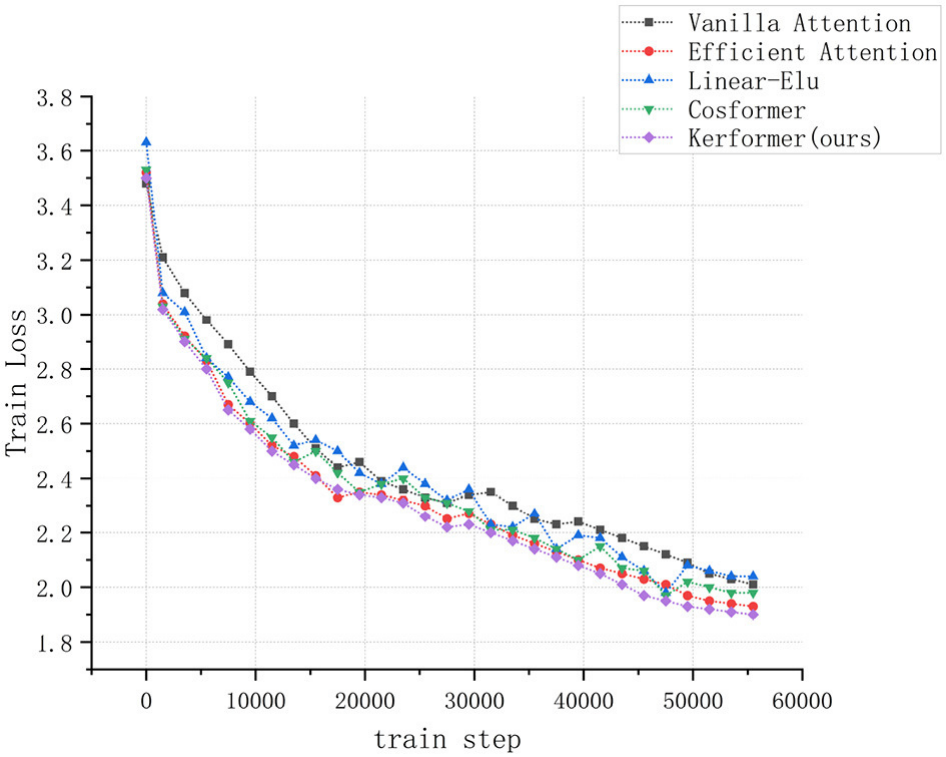
Comparison of convergence speed of ViT models using Vanilla attention, Efficient attention, Linear Elu, Cosformer and Kerformer when trained on the CIFAR-10 dataset.

**Figure 8:**
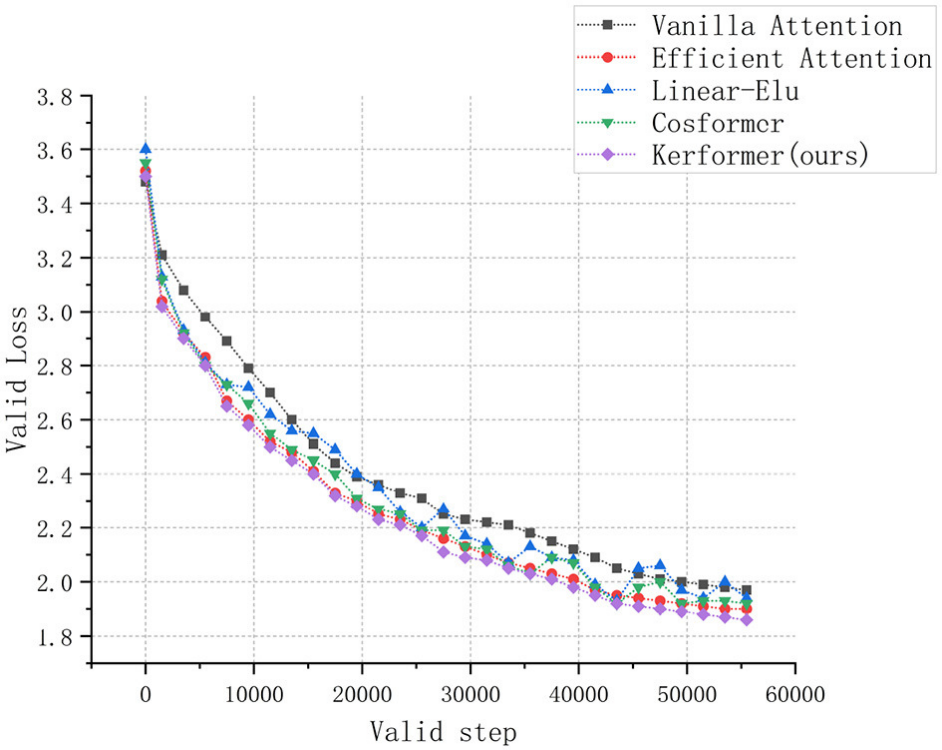
Comparison of convergence speed of ViT models with Vanilla attention, Efficient attention, Linear Elu, Cosformer and Kerformer when validated on CIFAR-10 dataset.

In addition to evaluating the model performance and running cost, we also conducted experiments to measure the convergence speed of the ViT model during training and validation on the CIFAR-10 dataset using three methods: Vanilla attention (Vaswani et al., 2017), Efficient attention (Shen et al., 2021), Linear Elu (Katharopoulos et al., 2020), Cosformer (Qin et al., 2022), and our proposed Kerformer. The results of these experiments are presented in Figures 7, 8.

The experimental results demonstrate that our proposed method can achieve a faster convergence rate compared to the other four methods, Vanilla attention, Efficient attention, Linear Elu and Cosformer, in the training and validation of the ViT model on the CIFAR-10 dataset. This result fully demonstrates the effectiveness of our proposed method in reducing the training cost of the model.

Compared to traditional attention mechanisms, our proposed improvement achieves better results with less computational cost, indicating that our method can train better models in less time. Therefore, our proposed method has better efficiency and higher performance, making it an effective attention mechanism improvement scheme.

Kerformer provides a good idea of linear complexity by linearizing attention by the operation of activating the *Q* and *K* matrices and reweighting the activated K matrices can effectively maintain linear complexity with guaranteed effective attention. In the experimental results Kerformer did not perform best on all tasks, which may be due to the specific nature of the task or the fact that some tasks require a special model structure resulting in poor performance of Kerformer on that task. Also the characteristics of the dataset, the experimental setup, and the choice of hyperparameters may have affected the experimental results of Kerformer on this task.

## 7. Conclusion

We propose a new Kerformer method to linearize the attention mechanism by the kernel function method to first process the *Q* and *K* matrices non-negatively, then reweight the non-negatively processed *K* matrices by SE Block to amplify the localization relation of the attention matrix, and finally change the order of operations of the attention matrix by the combination law of matrix operation to convert Transformer's computation of the complex attention mechanism into a linear computation based on the sequence length *N*. We conducted experiments on text classification, Long-range arena, the computational speed of the model on long sequences, and on image classification, respectively, and the experimental results show that Kerformer performs well on these different tasks. This well demonstrates that the Kerformer model can exhibit good model performance and computational efficiency both on NLP tasks and on image tasks, which can make Kerformer widely applicable to different fields where attention mechanisms exist. Overall, our approach can achieve high model performance with low running cost, which allows the deployment of models with attention mechanisms to some devices with low computational power.

In the future, we hope that our proposed method can be widely applied to the computational process of attention mechanism to reduce the running cost of the model, and we will continue to optimize our method so that it can be widely applied to different downstream tasks.

## Data availability statement

Publicly available datasets were analyzed in this study. This data can be found at: https://www.cs.toronto.edu/~kriz/cifar.html; http://yann.lecun.com/exdb/mnist/; https://www.tensorflow.org/datasets?hl=zh-cn.

## Author contributions

YG designed the research project, conducted experiments, analyzed the data, and wrote the paper. YF and YL provided guidance and feedback on the research design, data analysis, and paper writing. DW helped with data collection, experiment design, and manuscript proofreading. All authors have read and approved the final manuscript.
